# A systematic review of pre-registration in autism research journals

**DOI:** 10.1177/13623613241308312

**Published:** 2024-12-25

**Authors:** Daniel Poole, Audrey Linden, Felicity Sedgewick, Oliver Allchin, Hannah Hobson

**Affiliations:** 1 University of Sheffield, UK; 2 Open University, UK; 3 University College London, UK; 4 University of Bristol, UK; 5 University of York, UK

**Keywords:** autism, autism research, meta-research, pre-registration

## Abstract

Pre-registration refers to the practice of researchers preparing a time-stamped document describing the plans for a study. This open research tool is used to improve transparency, so that readers can evaluate the extent to which the researcher adhered to their original plans and tested their theory appropriately. In the current study, we conducted an audit of pre-registration in autism research through a review of manuscripts published across six autism research journals between 2011 and 2022. We found that 192 publications were pre-registered, approximately 2.23% of publications in autism journals during this time frame. We also conducted a quality assessment of a sample of the pre-registrations, finding that specificity in the pre-registrations was low, particularly in the design and analysis components of the pre-registration. In addition, only 28% of sampled manuscripts adhered to their analysis plan or transparently disclosed all deviations. Autism researchers conducting confirmatory, quantitative research should consider pre-registering their work, reporting any changes in plans transparently in the published manuscript. We outline recommendations for researchers and journals to improve the transparency and robustness of the field.

**Lay abstract:**

When researchers write down their plans for a study ahead of time and make this public, this is called pre-registration. Pre-registration allows others to see if the researchers stuck to their original plan or changed as they went along. Pre-registration is growing in popularity but we do not know how widely it is used in autism research. In this study, we looked at papers published in six major autism journals between 2011 and 2022. We found that only 2.23% of papers published in autism journals had been pre-registered. We also took a close look at a selection of the pre-registrations to check how good they were and if researchers stuck to their plans. We found that the pre-registrations generally lacked specifics, particularly about how the study was designed and the data would be analysed. We also found that only 28% of the papers closely followed the pre-registered plans or reported the changes.

Based on these findings, we recommend that autism researchers consider pre-registering their work and transparently report any changes from their original plans. We have provided some recommendations for researchers and journals on how pre-registration could be better used in autism research.

## Introduction

The field of autism research spans a range of topics and methodological approaches, but confirmatory, quantitative studies have constituted most published work (Bölte, 2014). Broadly, autism researchers using quantitative methods work within a hypothetico-deductive framework whereby a hypothesis is deduced from a theory about autism which is subsequently tested in an empirical study. The researcher then evaluates whether the study provides evidence for the theory. Through the accumulation of studies, theories (about autism) can then either be retained or discarded (Fidler et al., 2018; Popper, 1959).^1^

In the wider scientific literature, attention has been drawn to the use of questionable research practices which have likely inflated the rate of false-positive findings (i.e. reporting the presence of an effect when in reality none exists; Ioannidis, 2005). A set of strategies referred to as researcher degrees of freedom describe how researchers can run many statistical tests and/or try out different data decisions before selectively reporting only those which yielded a ‘statistically significant’ result (Simmons et al., 2011). Strategies which have been described as researcher degrees of freedom include (a) optional stopping where the researcher runs unreported interim data analysis and stops data collection once statistical significance is reached and (b) the selective rejection of data points and variables based on statistical results (Head et al., 2015; Simmons et al., 2011). Researchers who use frequentist statistics with the criterion of α < 0.05 for statistical significance typically accept a false-positive rate of 1 in 20. Running multiple tests in this way undermines this assumption and the likelihood of false positives increases rapidly (see Stefan & Schönbrodt, 2023, for a systematic investigation of the consequences of different researcher degrees of freedom on false-positive rates). Closely related is Hypothesising After Results Are Known (HARKing), which refers to the post hoc construction of a hypothesis following the results of statistical tests (Kerr, 1998).

When engaging in these questionable research practices, researchers are presenting exploratory research as though they were confirmatory (Wagenmakers et al., 2011). This undermines the process of knowledge generation through the hypothetico-deductive framework which relies on the assumption that hypotheses are generated from theory, independently of the data used to test it. Philosophers of science have suggested that theories should be retained based on withstanding risky tests (Mayo, 1983, 1991; Meehl, 1990). In this context, ‘risky tests’ refers to studies which are designed in such a way that they will be very likely to produce a negative finding if the prediction is wrong. Where researchers engage in questionable research practices, they are using increasingly risk free tests, meaning that the theories are not being meaningfully assessed. Surveys of academic researchers have suggested that questionable research practices are widespread across the biomedical and social science fields (John et al., 2012; Martinson et al., 2005; Xie et al., 2021). Furthermore, when making data-dependent analysis decisions, researchers can be engaging in questionable research practices inadvertently. That is, the researcher may not consider themselves to be ‘fishing’ by actively seeking positive findings through running multiple tests, but they are exposed to the risks of increased researcher degrees of freedom when their analysis plan is shaped by looking at the data (Gelman & Loken, 2013).

Pre-registration has emerged as a popular solution to reduce questionable research practices and better constrain researcher degrees of freedom (Nosek et al., 2018). A pre-registration is a time-stamped protocol that outlines the researcher’s planned approach, created before the study begins and published alongside the study’s results. Pre-registration is a way of improving the readers’ trust that the research was confirmatory rather than exploratory (Nosek et al., 2019; Wagenmakers et al., 2012) and enables them to evaluate the riskiness of the test (Lakens, 2019; Lakens et al., 2024).

There are a number of varieties of pre-registration (see Hardwicke & Wagenmakers, 2023, for an overview and historical context). Pre-registration was popularised through clinical trials as an attempt to safeguard against publication bias and selective reporting (although notably the requirements for clinical trial registration do not extend to the registration of analysis plans). From 2005, the International Committee of Medical Journal Editors required that clinical trial protocols were publicly pre-registered prior to recruitment as a condition of publication (De Angelis et al., 2005).^2^ Following this requirement, a study of cardiovascular interventions found that the rate of positive results reported in studies prior to 2000 (none pre-registered) was 57% dropping to 8% after 2000 (all pre-registered; Kaplan & Irvin, 2015).

A number of registries for hosting pre-registration emerged, following the publication of high-profile papers highlighting that questionable research practices and false positives were likely widespread across research fields (Begley & Ellis, 2012; Ioannidis, 2005; Simmons et al., 2011). This includes registries for general pre-registration such as the Open Science Framework and AsPredicted. There are also registries which are field specific, or for specific methods. For example, the International Prospective Register of Systematic Reviews (PROSPERO) is the registry for systematic review and meta-analysis protocols. The Preferred Reporting Items for Systematic Reviews and Meta-Analysis (PRISMA) statement recommends that systematic reviewers provide an identifier for a prospectively registered protocol with the completed review (Liberati et al., 2009; Moher et al., 2009; Page et al., 2021).

From 2013, select journals began to offer Registered Reports, which is a form of pre-registration in which the authors submit the introduction and methods for a planned study to a journal where it is peer reviewed and can receive in principle acceptance. The authors then collect the data and write the analysis and discussion. In addition to constraining researcher degrees of freedom, this approach also safeguards against publication bias (Lakens et al., 2024). There is evidence that the rate of positive findings is reduced when using the Registered Reports format. Positive results were reported in 44% of psychology/psychiatry studies published between 2013 and 2018, compared with 96% of articles taken from a comparable sample using the standard manuscript format (Scheel et al., 2021).

As a largely confirmatory, quantitative field, questionable research practices likely impact the autism research literature.^3^ Until recently (Hobson et al., 2022, 2023; Sandbank et al., 2024), there has been little consideration of how open research practices (such as pre-registration) are used in the context of autism research. Meta-research studies investigating autism interventions have found that study quality is low (Bottema-Beutel et al., 2023) and that there is a lack of transparency in how conflicts of interests have been reported (Bottema-Beutel & Crowley, 2021). Indeed, many autistic people distrust researchers (Botha, 2021; Gowen et al., 2019; Pellicano et al., 2014) and there is a dissatisfaction that the spending on autism research has not translated into meaningful impact on the lives of autistic people and their families (Pellicano et al., 2014b). Although not a panacea for these problems, as discussed above, pre-registration makes the research process more transparent, test of hypotheses riskier and may reduce the rate of false positives in the field.

In the present study, we aimed to better understand how pre-registration has been used in autism research. To date, there has not been a systematic investigation into the use of open research methods in autism research journals. In the current study, we reviewed publications in six autism research journals (*Autism, Autism in Adulthood, Autism Research, Journal of Autism and Developmental Disorders, Molecular Autism, Research in Autism Spectrum Disorders)* between 2011 and 2022 to estimate the prevalence of pre-registration in the field. We selected these journals as the six highest impact factor autism specialist journals (Clarivate, 2022). The date range was selected as 2011 was a year when several seminal studies drew attention to the issue of research degrees of freedom (e.g. Open Science Collaboration, 2012; Simmons et al., 2011; Wagenmakers et al., 2011) and domain general registries for pre-registration emerged in the following years (Nosek et al., 2018). In addition, we conducted a quality review on a sample of the pre-registered studies using pre-existing tools. We looked at the extent to which the pre-registrations were specified and appropriately constrained the researcher’s degrees of freedom (Bakker et al., 2020). We also investigated the adherence to the pre-registration, and transparent reporting of any deviations, in the published manuscript (Claesen et al., 2021).

## Methods

### Search strategy

**Figure 1.**
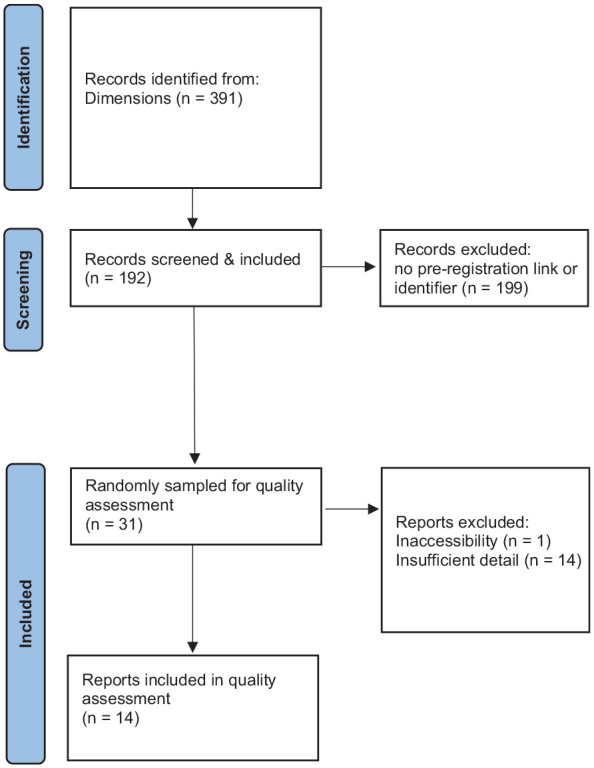
Flow diagram representing the selection of manuscripts.

The search strategy was pre-registered (https://osf.io/yqrjh) and conducted in accordance with the guidance in the PRISMA statement (Page et al., 2021). Following consultation with a librarian, we used Dimensions (Hook et al., 2018) as full-text search is available for ~70% of indexed publications, which is useful for identifying links to pre-registrations in the body of the manuscript. Dimensions has excellent journal coverage (Singh et al., 2021). In addition, Boolean searches are possible with Dimensions meaning that the search is precise and reproducible. The search terms were preregistration OR preregister OR pre-registration OR pre-register OR OSF OR ‘Open Science Framework’ OR aspredicted OR PROSPERO with the following filters: Date: 2011–present. Source Title: *Autism, Autism in Adulthood, Autism Research, Journal of Autism and Developmental Disorders, Molecular Autism, Research in Autism Spectrum Disorders*. The final search was conducted on 29 November 2022. There were no exclusion criteria, and each full-text manuscript returned from this search was included in the final review. As only one database was searched, no duplicates were returned. See Figure 1 for the flow diagram of manuscript selection. We also searched Dimensions without the search terms so that we could estimate the number of pre-registered studies as a percentage of the total number of manuscripts. The results of this search indicated that 8597 were published in the target journals between 2011 and 2022.

### Data extraction

Each manuscript was hand searched for the terms ‘pre-reg’, ‘prereg’, ‘protocol’, ‘osf’, ‘open science’, ‘register’, ‘registration’, ‘aspredicted’ and ‘as.predicted’. Any article that included a link to a pre-registration or an identification code for a pre-registration protocol (e.g. a ClinicalTrials.gov NCT number) was coded as pre-registered. Where there was a link to an OSF page (for instance, where the study included open data), these were also checked. For manuscripts coded as pre-registered, the study design was classified as Systematic Review, Intervention, Observational, Experimental, Secondary Analysis or Qualitative based on the description in the title and abstract, with reference to the full text where required.

### Quality assessment

We conducted a quality assessment of a randomly selected sample of 20% of the manuscripts which reported empirical research (i.e. excluding systematic reviews) which were coded as ‘yes’ for including a pre-registration. Selection of manuscripts was conducted using the random number generator in R. The quality assessment was pre-registered (https://osf.io/87g2x). Two coders (D.P. and A.L. or H.H. or F.S.) independently coded each pre-registration and the associated manuscript. None of the researchers coded a manuscript that they authored. As described below, pre-registrations which did not reach the threshold for accessibility and minimum detail were not reviewed further. Once the first sample of manuscripts were coded, we resampled the remaining manuscripts until we were able to review our target sample size. Cohen’s kappa was calculated as a measure of agreement between coders. Discrepancies were resolved through discussion where scores were finalised via consensus with the reported analysis based on these finalised scores.

### Accessibility and minimum detail

Manuscripts were coded on accessibility and minimum detail using the scoring system described in Claesen et al. (2021). First, the pre-registration received an accessibility score of 0–6, with ratings of 0 or 1 on six items: permanent, read-only, time stamped, public, non-ambiguously accessible and in a third-party repository. Manuscripts scoring <6 were not reviewed further. Next the pre-registration was reviewed and received a minimum detail score of 0–6, with ratings of 0 or 1 on six items for containing information on hypothesis, dependent/independent variables, dependent/independent variable operationalisation, sample size, procedure and analysis plan. Pre-registrations scoring <6 were not reviewed further.

### Researcher degrees of freedom

Studies which reached the thresholds for accessibility and minimum detail were then further reviewed. First, pre-registrations were assessed for the extent to which researcher degrees of freedom were constrained using the tool provided by Bakker et al. (2020). This tool contains 22 questions in order to assess specificity across 29 possible researcher degrees of freedom across hypothesising, design, data analysis and reporting through review of the pre-registration document. Following the recommendation described in Bakker et al. (2020), we removed items that assessed power analysis (D6), random assignment (C1) and blinding (C2) as these are measures of study quality rather than specificity. Each researcher degree of freedom receives a specificity rating between 0 and 3 where increasing rating means greater specificity: 0 (not specified), 1 (partially specified), 2 (specific and precise) and 3 (specific, precise and exhaustive, that is, including a specific statement that the researcher will not deviate from their plans). There were items with less gradation, with scores of 1 not available for T1 (Hypothesis), T2 (Direction Hypothesis), D1 (Multiple Manipulated IVs), D3 (Multiple Measures DVs), A2 (Data Preprocessing), A5 (Select DV Measure), A9 (Operationalising Manipulated IVs) and R6 (HARKing). In addition, scores of 1 or 2 were not available for D4 (Additional Constructs), A7 (Select Primary Outcome), A8 (Select IV) and A10 (Include Additional IVs). Scores for each section were summed for each manuscript and a mean calculated across manuscripts.

### Discrepancies

We also assessed the extent to which any discrepancies between the pre-registration document and the manuscript were transparently reported using the tool provided by Claesen et al. (2021). Each pre-registration and published manuscript were reviewed across six items: hypothesis/research question, variables, sample size, exclusion criteria, procedure and analysis. Each item received a qualitative rating of ‘‘no deviations’, ‘all deviations disclosed’ or ‘undisclosed deviations’.

### Analysis

All data preparation, analysis and plotting of figures was conducted in R (version 4.3.2.) using the *tidyverse* package (Wickham et al., 2019); additionally, the *janitor* package (Firke, 2023) was used for data cleaning. The *itt* package (Gamer & Lemon, 2019) was used for calculating Cohen’s Kappa.

For a random sample of 20% of the manuscripts, the classification of the study design was independently second coded by HH. An unweighted Cohen’s Kappa was calculated as a measure of inter-rater reliability (ϰ = 0.85, *z* = 6.92, *p* < 0.001) showing excellent agreement between coders.

The total count of pre-registered manuscripts and a breakdown by classification of the study design were calculated. We also used data acquired by searching Dimensions with the search terms excluded to estimate the percentage of publications which were pre-registered by publication year and by journal.

### Quality assessment

For the researcher degrees of freedom, the weighted Cohen’s Kappa (suitable for ordinal measures) was suggestive of ‘substantial’ agreement between reviewers (ϰ = 0.621, *z* = 11.30, *p* < 0.001). We calculated descriptive statistics for each of the 26 rated researcher degrees of freedom. In addition, we plotted an average specificity score for each section and presented this in a tile plot.

For the discrepancy assessment, unweighted Cohen’s Kappa suggested ‘moderate’ reliability between the coders (ϰ = 0.584, *z* = 6.78, *p* < 0.001). Ratings by section were plotted for individual studies in a tile plot and by methodological aspect in a stacked bar plot.

### Deviations from pre-registration

We made the following deviations from our pre-registered procedure. F.S. was unavailable for much of the second coding, so O.A. and A.L. were included as second coders.

There were two manuscripts which we did not review despite the pre-registration reaching the accessibility and minimum detail threshold. These manuscripts included a link to a pre-registration, but these were for larger research projects and not the analysis described in the manuscript. We felt that including these studies would distort the measurement of the discrepancy review.

In our pre-registered protocol, we stipulated that we would randomly resample until reaching our target sample. In our initial sample, 10 of the 12 intervention studies did not reach the threshold for including sufficient detail for the quality assessment.^4^ When resampling, we decided to exclude intervention studies. We also adjusted the target sample size to *n* = 13, which was 20% of the remaining manuscripts (i.e. excluding interventions).

Finally, in addition to reporting the mean for each degree of freedom as pre-registered, we provide a tile plot showing the mean by section in order to visualise the specificity scores.

## Results

Raw data, analysis code and a master review documents are available on the study OSF page (https://osf.io/mjy4p/).

### Prevalence of pre-registrations in autism journals

**Figure 2.**
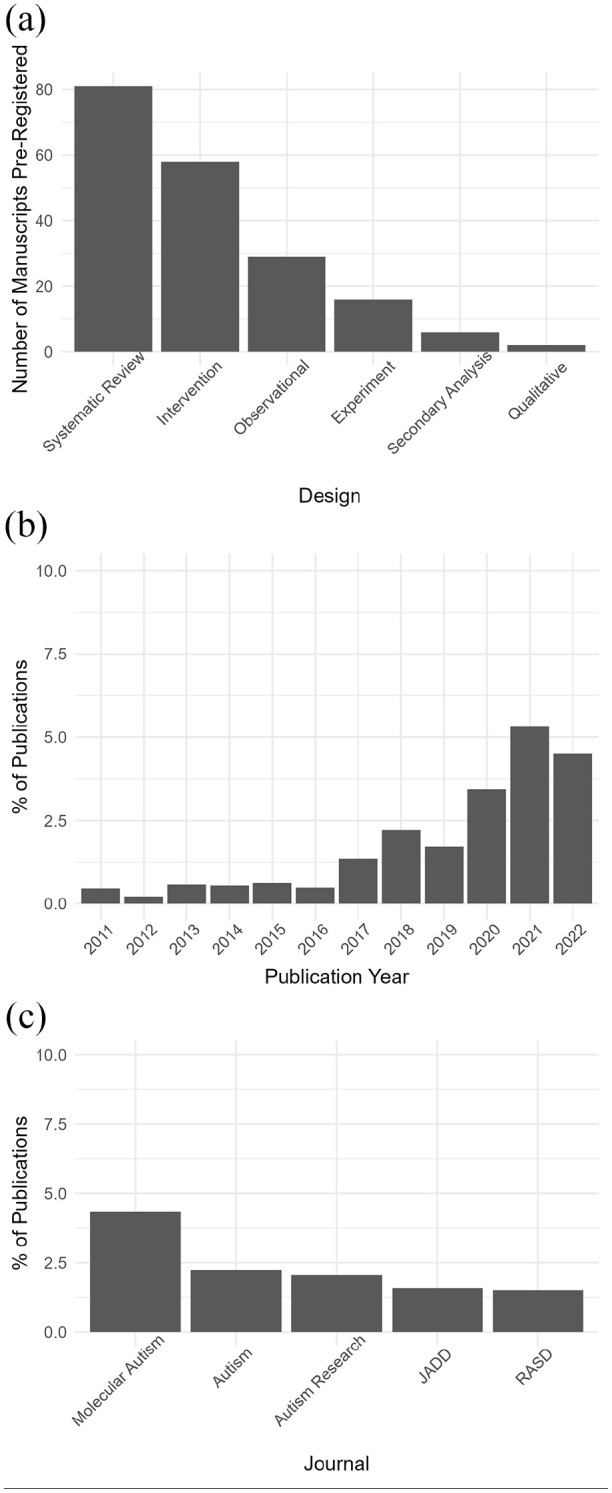
Breakdowns of manuscripts which were coded as pre-registered (a link or identification code to a pre-registration was in the manuscript). (a) Total number of manuscripts organised by type. (b) Percentage of manuscripts which included a pre-registration which were published across the journals each year. (c) Percentage of manuscripts which included a pre-registration across the time frame by journal (there were no pre-registrations in manuscripts published in *Autism in Adulthood*).

A total of 192 manuscripts were pre-registered. A breakdown of the record by year, by publication and by article type is included in Figure 2. Systematic reviews and intervention studies had the largest count of pre-registrations. There has been a general trend towards an increase in the percentage of publications including pre-registrations since 2017. The journal *Molecular Autism* had the greatest percentage of published work that was pre-registered.

### Quality assessment

#### Accessibility

In total, 31 manuscripts were sampled. All but one pre-registration reached the threshold for accessibility:^5^ in this instance, the identification code provided in the manuscript was not linked to a pre-registration we could locate.

#### Minimum detail

**Figure 3.**
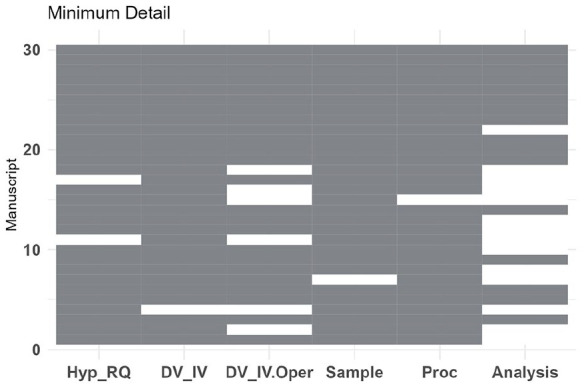
Tile plot displaying the ratings for minimum detail for the 31 manuscripts which were assessed. Pre-registrations were rated for containing minimum detail about the study Hypothesis and Research Question (Hyp_RQ), Dependent and Independent Variables (DV_IV), Operationalisation of the DV and IV (DV_IV.Oper), planned sample size or stopping rule (Sample), Procedure and Analysis. Where the pre-registration scored <6, that manuscript was not reviewed further.

Fourteen of the pre-registrations did not reach the threshold for including minimum detail to review further. Six pre-registrations scored 5 for not including an analysis plan, with the remaining 8 not reaching the threshold for minimum detail across multiple sections. The scores for individual manuscripts are presented in Figure 3. Manuscripts which were rejected for insufficient detail were interventions (*n* = 10), observational (*n* = 3) and secondary analysis (*n* = 1).

Fourteen studies received full quality assessment (see Table 1).

**Table 1.** List of studies included in the quality assessment.

Study	Year	Journal	Type
1	2019	*JADD*	Experiment
2	2020	*Autism Res*	Intervention
3	2021	*JADD*	Observational
4	2022	*JADD*	Secondary analysis
5	2022	*RASD*	Observational
6	2021	*Autism Res*	Intervention
7	2022	*Autism*	Experiment
8	2022	*Autism*	Observational
9	2022	*JADD*	Experiment
10	2021	*Autism*	Observational
11	2020	*JADD*	Observational
12	2021	*JADD*	Observational
13	2021	*Autism Res*	Experiment
14	2022	*JADD*	Observational

Researcher degrees of freedom: descriptive statistics of specificity score by the 26 researcher degrees of freedom are given in Table 2. Items with a mean rating <1 were additional IVs (D2), data handling/collection (C3), missing data (A1), assumptions (A3), additional IVs (A10), method and package (A14) and inference criteria (A15). Items with a mean rating of 0 were multiple manipulated IVs (D1), additional constructs (D4), select primary outcome (A7) and HARKing (R6).

**Table 2.** Mean, standard deviation (SD), range and count of NAs for each degree of freedom.

	DoF	Mean (SD)	Range	NA
Hypotheses				
	T1: Hypothesis	1.60 (0.85)	0–2	0
	T2: Direction hypothesis	1.35 (0.93)	0–2	0
Study design				
	D1: Multiple manipulated IVs	0 (0)	0–0	9
	D2: Additional IVs	0.21 (0.80)	0–3	0
	D3: Multiple measures DV	2	0–2	0
	D4: Additional constructs	0 (0)	0–0	0
	D5: Adding exclusion variables	1.64 (1.01)	0–3	0
	D7: Sampling plan	1.28 (0.47)	1–2	0
Data collection				
	C3: Data handling/collection	0.61 (0.77)	0–2	1
	C4: Stopping rule	1.21 (0.58)	0–2	0
Analysis				
	A1: Missing data	0.71 (0.73)	0–2	0
	A2: Data preprocessing	2	2–2	14
	A3: Assumptions	0.36 (0.63)	0–2	0
	A4: Outliers	1(1.11)	0–3	0
	A5: Select DV measures	1.86 (0.53)	0–2	0
	A6: DV Scoring	1.07 (0.64)	0–2	1
	A7: Select primary outcome	0	0–0	0
	A8: Select IV	0.29 (0.76)	0–2	8
	A9: Operationalising manipulated IVs	1 (1.06)	0–2	7
	A10: Include additional IVs	0.23 (0.83)	0–3	1
	A11: Operationalising non-manipulated IVs	1.50 (0.71)	0–2	4
	A12: In/Exclusion criteria	1.64 (1.01)	0–3	0
	A13: Statistical model	1.43 (0.65)	0–2	0
	A14: Method and package	0.21 (0.42)	0–1	0
	A15: Inference Criteria	0.79 (0.81)	0–2	0
Reporting				
	R6: HARKing	0	0–0	0

Scores could range between 0 and 3 with higher scores indicating higher specificity. SD: standard deviation.

**Figure 4.**
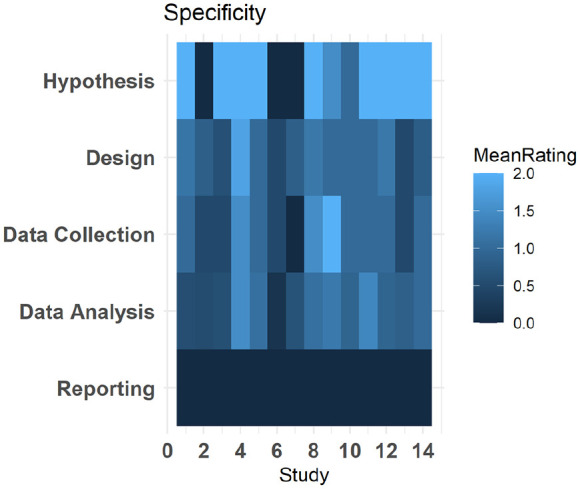
Tile plot displaying the average specificity score across researcher degrees of freedom by section Hypothesis, Design, Data Collection, Data Analysis and Reporting. Increasingly light shades of blue indicate greater specificity in that section. Note comment in Discussion section regarding scores of 0 for Reporting.

In addition, the mean rating by section (Hypothesis, Design, Data Collection, Data Analysis and Reporting) for each manuscript is provided in Figure 4.

### Adherence

**Figure 5.**
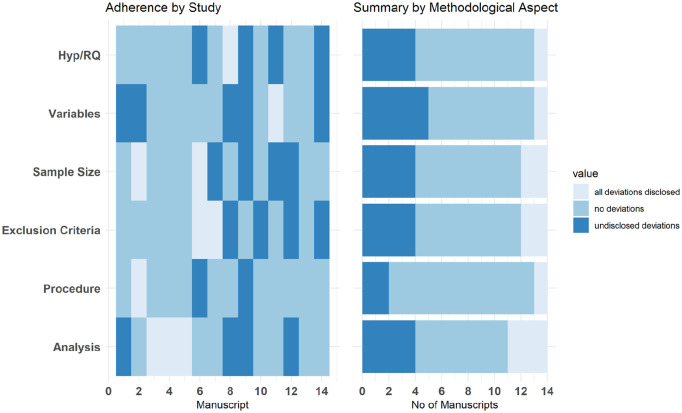
Tile Plot (left) and stacked bar chart (right) showing deviations (none, disclosed and undisclosed) by section Hypothesis/Research Question (H.RQ), Variables, Sample Size, Exclusion Criteria, Procedure and Analysis.

The adherence ratings for individual manuscripts and a summary are provided in Figure 5. One manuscript did not deviate from the pre-registration and three manuscripts transparently disclosed all deviations.

Undisclosed deviations were most common for variables (*n* = 5) and lowest for procedure (*n* = 1).

## Discussion

While it has been argued that pre-registration could help address issues of unconstrained researcher degrees of freedom and thereby improve reproducibility, no systematic study of the uptake and application of this approach to autism research had taken place. We identified 192 manuscripts that were published between 2011 and 2022 in the journals *Autism, Autism in Adulthood, Autism Research, Journal of Autism and Developmental Disorders, Molecular Autism* and *Research in Autism Spectrum Disorders*, which included a link to a pre-registration or an identification code. Our estimate of the prevalence of pre-registration in autism research journals is ~2.23%. This indicates that across the last decade, a time in which pre-registration has been promoted as an important tool to improve the reliability and robustness of research (Hardwicke & Wagenmakers, 2023; Nosek et al., 2018, 2019), it has been rarely used in the field.

There have been previous attempts to estimate the prevalence of pre-registration as part of the study of open research practices in other disciplines. Hardwicke et al. (2022) sampled psychology manuscripts (as based on PubMed identification numbers) published between 2014 and 2017 and rated a randomly selected sample of manuscripts on the use of open research practices. The use of pre-registration was estimated at 3% (5/188 manuscripts were pre-registered). A study using the same method for social science research estimated pre-registration prevalence at 0% (0/156 manuscripts, Hardwicke et al., 2020). In addition, estimates from studies which have used a different method for selecting manuscripts for gambling research published between 2016 and 2019 was 1.6% (8/500, Louderback et al., 2023) and linguistics 2008–2009 and 2018–2019 was 0% (0/519, Bochynska et al., 2023). This suggests that, although our estimate of the prevalence of pre-registration in autism research journals is very low, it is comparable to estimates from other fields. It is important to recognise that while these comparisons offer context for the prevalence estimate in the current study, the variation in practice between fields prevents meaningful direct comparison. For instance, pre-registration is more widely used in intervention studies and systematic reviews (as observed in the present study), likely as a consequence of explicit instructions that pre-registration is a component of the method, and the extent to which these methods are common in the field will shape the estimated prevalence of pre-registration. In addition, the methods used in estimating prevalence vary between studies, in particular relating to the date range and sampling approach. For the current study, we should also note constraints on the comparisons between journals for similar reasons. For instance, *Autism in Adulthood* has only been accepting publications since 2019 and publishes more qualitative research.

In the current study, we also conducted a quality assessment of a random sample of the pre-registrations. Pre-registrations were assessed for accessibility and minimum detail. All but one manuscript was accessible, but 14 did not reach the threshold for minimum detail. The previous study which used this tool observed seven studies (18% of the sample) not proving minimum detail (investigating studies awarded the pre-registration badge at *Psychological Science*, Claesen et al., 2021). Here, 10 of the studies which did not include minimum details were intervention studies which had been registered on trial registries (such as clinicaltrials.gov). We note that these registries do not include any specific questions about the analysis plans of the study. This likely reflects that the primary goal of pre-registering intervention studies on trial registries is to reduce publication bias (Hardwicke & Wagenmakers, 2023). Nonetheless, with the emergence of repositories for ‘standard pre-registration’, researchers could include a link from the registry to a detailed analysis plan.

We also reviewed the specificity of the pre-registrations, that is, the extent to which the pre-registration successfully constrained the researcher degrees of freedom (Bakker et al., 2020). The mean rating of items relating to analysis (5 degrees of freedom) and design (3 degrees of freedom) were lower than one, indicating that these researcher degrees of freedom were on average (less than) partially specified. Similarly, these items were among the lowest rated in previous studies which used this tool (Bakker et al., 2020; Heirene et al., 2021). The specificity rating for reporting was 0 for each item in the sample (with similar ratings reported by the previous studies using this tool). However, it is important to note that the rating for reporting relates to only a single degree of freedom (R6, HARKing), and this item is coded according to specificity in the hypothesis (Q1) and the text in the pre-registration explicitly stating that no other dependent variables apart from those tested in the hypothesis will be tested (Q7). It is reasonable to assume that researchers see the use of the pre-registration as restricting the use of additional dependent variables without needing to explicitly state this (a similar point was noted by Bakker et al., 2020). As such we suggest that this low rating is reflective of the stringency of the tool rather than an issue with autism researchers under specifying their hypothesis in pre-registrations. Indeed, the discrepancy review revealed that only four manuscripts had undisclosed deviations in the hypothesis between pre-registration and manuscript.

Finally, we assessed adherence to the pre-registration in the published manuscript. Four manuscripts either did not deviate from the pre-registration or transparently disclosed all deviations (28% of the sample). Undisclosed deviations were observed at a similar level across all sections in the manuscripts, apart from the procedure section where only a single manuscript was coded for undisclosed deviations. In the previous study which used this tool to assess manuscripts which were published with a pre-registration badge in Psychological Science between 2015 and 2017, 18% of the sample did not deviate from the pre-registered plans or transparently disclosed any deviations (Claesen et al., 2021). Undisclosed deviations were most common for the analysis and exclusion criteria in this study. A common misconception around pre-registration is that researchers are trapped by decisions they made before beginning the study, whereas deviations from the pre-registered plan may often be desirable and improve the quality of the research (Hardwicke & Wagenmakers, 2023; Lakens et al., 2024; Nosek et al., 2019). However, it is important that any deviations are transparently reported so that the riskiness of the test can still be evaluated. Lakens (2024) identified unforeseen events, mistakes, missing information/low specificity in the pre-registration, requiring unanticipated removal of data points and falsification of assumptions as reasons why researchers might need to deviate from their pre-registered plans. In each instance, the recommendation is that the deviation is explained and the possible consequences of the deviation considered. Furthermore, a template for reporting deviations in a systematic and transparent way has recently been provided (Willroth & Atherton, 2024) whereby the deviations are listed in a table including the wording in the pre-registration and manuscript, plus the possible implications of the change.

In summary, the current work has shown that it is rare for work published in autism research journals to include a pre-registration. In addition, of those studies that were sampled which included a pre-registration, our quality assessment has highlighted improvements in the pre-registration and reporting in the manuscript. In particular, the specificity in the pre-registration, particularly in the components describing the study design and analysis, and the deviations from the pre-registered plan could be reported in the manuscript more transparently. We offer further reflections and recommendations for autism researchers and journal editors arising from this review before considering the limitations of our work.

### Recommendations for researchers

Recent years have seen the emergence of practices to improve the reliability of confirmatory research (Munafò et al., 2017). Many autistic people find participating in research to be a challenging and stressful experience (Gowen et al., 2019), so it is especially important that their contribution is to research which is most likely to be robust and valid. We note that our own understanding of pre-registration has improved considerably from engaging in the quality assessment reported here. As such, we recommend that autism researchers’ training on using (and evaluating) pre-registration effectively might involve reviewing pre-registrations using the tools provided by meta-researchers similar to those we have used here. However, completing the quality assessments of pre-registrations was time consuming and cognitively demanding. As described by Claesen et al. (2021) in relation to the psychology literature, identifying deviations was challenging due to changes in formatting and terminology between pre-registration and manuscript. To help peer reviewers and fellow researchers, it would be a benefit if researchers aimed to present the hypothesis, variables and analysis as similarly as possible between the pre-registration and the manuscript.

When preparing pre-registrations, templates are commonly provided which provide prompts for details that the researcher should include. Templates are designed to be generalisable across research methods. However, the low specificity in researcher degrees of freedom observed here and in previous work (Bakker et al., 2020; Heirene et al., 2021; Van den Akker et al., 2023) suggest that further prompting on key issues might be useful. It could be that community designed modular templates, where the researcher can build a template which is suitable for their study design, would be useful. For instance, an autism researcher running an eye-tracking study could download a module with items relating to the inclusion of autistic participants (e.g. whether participants require a formal autism diagnosis, how the diagnosis might be confirmed, whether participants with co-occurring neurotypes or mental health conditions will be included) and a module with questions relating to the processing and analysis of eye-tracking data.

Finally, we encourage more transparent reporting of the study pre-registration. As noted in our discrepancy section, there were two studies which we decided not to review where the manuscript linked to a pre-registration for a larger study, not the work in the manuscript. In these instances, it was unclear why the link was included with the published manuscript. In addition, during the quality assessment, we noticed that five pre-registrations across those sampled were uploaded retrospectively (although neither tool we used for quality assessment included items about whether the registration was prospective or retrospective and this is an issue that may warrant focused investigation in future work). Where a study has been registered retrospectively, the reader can no longer evaluate whether the hypothesis and theory have been subjected to a risky test (Lakens et al., 2024). Researchers should clearly and unambiguously state whether the linked pre-registration is for the study described in the paper and whether it is a prospective or retrospective registration.

### Recommendations for journal editors

A number of journals award badges for engaging with open research practices, including a pre-registration badge awarded to manuscripts which provide a prospective pre-registration with transparent reporting of discrepancies (Open Science Framework, n.d.). Open research badges have been shown to increase the publication of studies using open research practices (Kidwell et al., 2016). Currently, no autism journals offer open research badges (see https://topfactor.org/) and pre-registration badges could be a simple intervention to encourage the adoption of the practice. However, issues with the badge system have been noted (Thibault et al., 2023), in particular that badges have been awarded without studies reaching the stated criteria and that resources are not being provided to peer reviewers in order to check the pre-registrations appropriately. *Psychological Science*, the first journal to introduce badges, has recently decommissioned them (Hardwicke & Vazire, 2023). At a minimum, autism journals could require either information from authors about pre-registration or an explanation of why they did not pre-register. Authors who provide a pre-registration could also be required to transparently and systematically report where they have deviated from the pre-registration (for instance using the template provided by Willroth & Atherton, 2024, mentioned above). Peer reviewers could be provided with tools to evaluate the pre-registration (such as those used in this study or alternatives such as TARG Meta-Research Group and Collaborators, 2022).

Another initiative would be for autism journals to engage with the publication of registered reports (Hobson et al., 2021). *Autism* and *Research in Autism Spectrum Disorder* have recently introduced the registered report format, although few have been published yet. Offering registered reports would provide an option for autism researchers to make use of a form of pre-registration which protects against both questionable research practices and publication bias. As the analysis is peer reviewed before the study begins, the registered report format can address issues around specificity and adherence. A recent initiative is Peer Community in Registered Reports (PCI-RR, n.d.) which organises the peer review of pre-prints in the registered report format. There are a number of PCI-RR friendly journals which commit to publishing based on these recommendations (i.e. with no further review). Autism journals could consider signing up to be PCI-RR friendly without having to commit to finding editors with expertise in managing registered reports. A final, perhaps counterintuitive recommendation is for autism research journals to consider offering Exploratory Reports (McIntosh, 2017). Exploratory reports are non-hypothesis-driven quantitative research where the focus is on exploring data, better characterising and developing measures or testing assumptions. This work can form the basis for later, more risky hypothesis testing. It has been noted of psychology that reducing the focus on confirmatory hypothesis testing in order to better develop concepts, measures and assumptions would benefit the field (Scheel, 2022; Scheel et al., 2021). In autism research, explicitly exploratory research, making use of participatory methods (see Hobson et al., 2023), could provide an opportunity to develop conceptually well-defined research paradigms which are valid and reflective of the priorities of autistic people which could later be tested in confirmatory research.

As a final point, it is important to note that there is a time cost to engaging in open research practices effectively, which are not appropriately accounted for in management workload models (see Hostler, 2024). As such, system level changes to support researchers are required to better enable changes in individual practices.

### Limitations

In this work, we focused on research published in autism journals, which does not provide a complete view of autism research. The estimated prevalence may have been shaped by our choice of search strategy. For instance, instead of focusing on autism research journals, we might have sampled autism research published across journals and make an estimate from there. Indeed, it may be that autism researchers are publishing pre-registered research in more generalised journals. In addition, in targeting these journals, we excluded manuscripts which were (a) not published in English and (b) not peer reviewed. The sample size of pre-registrations used in the quality assessment was small, meaning the quality assessment should be considered a preliminary investigation. We also acknowledge that the investigation of pre-registration is only informative about the riskiness of the testing in the individual studies and does not tell us anything about whether the findings will replicate or if they are likely to be true. Indeed, it has been noted that open research inventions have not been evaluated for the extent to which they improve reproducibility (Devezer et al., 2021; Szollosi et al., 2020).

## Conclusion

In the current study, we estimated the prevalence of pre-registration in the autism journals *Autism, Autism in Adulthood, Autism Research, Journal of Autism and Developmental Disorders, Molecular Autism*, and *Research in Autism Spectrum Disorders* between 2011 and 2022. We found that pre-registration was uncommon, only ~2.2% of the publications included a pre-registration. In addition, we completed a quality assessment of a sample of the pre-registered studies and found the specificity of the pre-registrations (to better constrain researcher degrees of freedom) and the reporting of discrepancies between the pre-registration and manuscript could be improved. We have highlighted recommendations that researchers and journals could do to encourage high-quality pre-registration which would improve the reliability of our findings and transparency in our field.

## Notes

1. It is important to note that research working in this framework is just one source of knowledge about autism. Autistic people’s own expertise (plus those of families and professionals), interpretative qualitative methods and exploratory research do not follow the hypothetico-deductive framework, but are all part of autism knowledge production.

2. Note also that the Declaration of Helsinki, which is widely cited as the guiding document of study ethical approval, includes article 35 stating that ‘Every research study involving human subjects should be publicly registered in a publicly accessible database before recruitment of the first subject’ (World Medical Association, 2013).

3. We note that pre-registration can also be relevant to qualitative analysis which is ‘scientifically descriptive’ (Finlay, 2021). However, for interpretative qualitative analysis, transparency and robustness are better achieved through researcher reflexivity and quantitative standards should not be inappropriately forced on qualitative methods (see Hostler, 2024; Steltenpohl et al., 2023, for detailed discussions).

4. Initially, an additional intervention study was rejected, but we subsequently reincluded this as the second coder had located a more detailed protocol.

5. In addition, one manuscript had the pre-registration link in the manuscript, but we were able to access the pre-registration via the first author’s Open Science Framework (OSF) page.
